# Misconceptions about transmission, symptoms and prevention of HIV/AIDS among adolescents in Ebonyi state, South-east Nigeria

**DOI:** 10.1186/s13104-020-05086-2

**Published:** 2020-05-14

**Authors:** Ifunanya Clara Agu, Chinyere Ojiugo Mbachu, Chinyere Okeke, Irene Eze, Chibuike Agu, Uchenna Ezenwaka, Nkoli Ezumah, Obinna Onwujekwe

**Affiliations:** 1 Health Policy Research Group, University of Nigeria, Enugu, Nigeria; 2 Department of Community Medicine, University of Nigeria, Enugu, Nigeria; 3 Department of Health Administration and Management, University of Nigeria, Enugu, Nigeria; 4 Department of Community Medicine, Ebonyi State University, Abakaliki, Nigeria

**Keywords:** Misconceptions, Adolescents, HIV/AIDS, Transmission, Prevention

## Abstract

**Objectives:**

Nigeria has the second largest number of adolescents and young people living with HIV/AIDS in the world. Misconceptions about HIV/AIDS contribute to spread of HIV, and constrain uptake of preventive services. This paper explored misconceptions about HIV/AIDS among adolescents in south-east Nigeria. A qualitative study was conducted in six urban and rural local government areas of Ebonyi state. Data were collected through twelve focus group discussions (FGD) with unmarried adolescents aged 13–18 who were either attending school or out-of-school. The FGDs were conducted using a pre-tested topic guide. Data were coded manually and analyzed using a thematic framework approach.

**Results:**

There are persistent misconceptions about transmission of HIV/AIDS through mosquito bites and sharing of personal belongings. Some adolescents had inaccurate notions that a HIV infected person could be identified through changes in physical features such as abdominal swelling and longer fingernails. A few of them also reported that HIV could be treated with antibiotics. These misconceptions were expressed by both male and female adolescents. Adolescents have some mistaken beliefs about HIV/AIDS which constrain them from taking necessary preventive measures. Hence, the need to target adolescents with health education interventions on HIV/AIDS.

## Introduction

Global estimates show that Nigeria has one of the highest numbers of HIV-infected adolescents worldwide [1]. As at 2016, 240,000 Nigerian adolescents aged 10–19 were living with HIV/AIDS [2], and studies have reported that many sexually active adolescents in Nigeria engage in risky sexual behaviours that increase their risk for HIV infection [3, 4]. Nigeria is making progress to scale up HIV/AIDS treatment and prevention services for adolescents and young people [5]. The National HIV/AIDS Strategy for Adolescents and Young People (2016–2020) and the National HIV and AIDS Strategic Framework (2019–2021) have been developed to accelerate and deliver a well-coordinated HIV response to all categories of adolescents and young people in Nigeria [6, 7].

Comprehensive knowledge on HIV/AIDS is fundamental for behavior change and uptake of HIV services because human behavior can be positively influenced by appropriate and complete information [8]. However, many adolescents in Nigeria lack comprehensive knowledge about HIV/AIDS; and are thus susceptible to misinformation and misconceptions about modes of transmission and methods of prevention of HIV [2, 9]. Misconceptions refer to scientifically proven wrong ideas and mistaken beliefs which are held by a group of people concerning a phenomenon [10]. These misconceptions are usually robust, resistant to change, deeply entrenched in peoples’ experiences, and interfere with individual learning [10]. Although efforts have been made, and are being made, to increase knowledge and awareness of HIV/AIDS, misconceptions about HIV prevention and transmission persist and are linked to risky sexual behaviors, including inability to negotiate safe sex, among young people [11, 12].

The tendency for information, including false information, to spread among young people is increasingly enabled by technological advancements and access to social media [13]. Evidence shows that most young people communicate through social media, and are very likely to believe information they receive from it. Considering the potentially harmful consequences of spreading mistaken beliefs about HIV through social media it is essential to explore these what these beliefs are, in order to understand what information about HIV needs to be debunked among young people. This paper examines misconceptions about modes of transmission, methods of prevention, and symptoms of HIV/AIDS among adolescents; as an important first step in designing health information interventions about HIV/AIDS.

## Main text

### Methods: study area

The study was conducted in Ebonyi state, south-east Nigeria, which is located on latitude 6°15′18″ N, longitude 8°05′55″ E, and shares a border with Benue state to the north, Enugu state to the west, Imo and Abia states to the south and Cross River state to the east. The state is inhabited mainly by people of Igbo tribe and has about 6 million inhabitants with over 40% of the total population under 15 years of age [14]. There are thirteen local government areas (LGAs) and three senatorial zones in the state.

### Study design and population

This was a qualitative cross-sectional study that explored perceptions of adolescents about the symptoms, mode of transmission, treatment and prevention of HIV/AIDS. The study population consisted of in-school and out-of-school unmarried adolescents aged 13 to 18 years who were living in the selected urban and rural areas.

### Sampling technique and data collection

A multi-stage sampling method was used to obtain the sample. At the first stage, six focal local government areas with reported high teenage pregnancy rates were purposively selected. At the second stage, one community with an established adolescent/youth friendly centre (to aid future project intervention) was selected from each local government area (LGA). Out-of-school adolescents who appeared to be more outspoken during the quantitative survey, were invited to participate in focus group discussions (FGD). In-school adolescents were selected at random from public secondary schools in the study communities.

In each community, two FGDs were conducted, one for boys and another for girls. A total of twelve FGDs were conducted using a pre-tested topic guide in English or Igbo language, according to participants’ preferences and audio recorded. Each FGD comprised of 8 to 10 participants and was facilitated by a moderator and a note taker. All adolescents gave written consent before participation. Parental consent was also obtained for adolescents less than 18 years. Information about the purpose of the study, and roles and rights of participants was provided prior to obtaining consent from parents and adolescents. Permission to audio record FGDs was obtained from participants. On average, each discussion lasted about 60–70 min.

### Data analysis

Audio recordings of interviews were transcribed verbatim and translated to English language, where necessary. Transcripts were compared with field notes to ensure completeness and inclusion of non-verbal responses. All transcripts were anonymised with codes and stored in password protected laptops. Data were analysed using a thematic framework approach. Themes were first generated from the study objectives and topic guide, and applied to two randomly selected transcripts (one male and one female FGD) independently by two researchers. Emerging themes and discrepancies in initial coding were reviewed by the research team. A final coding scheme/framework was generated and used to manually code all twelve transcripts. The thematic areas in the final coding scheme include, (i) perceptions about symptoms and signs of HIV; (ii) perceptions about modes of transmission of HIV; and (iii) perceptions about prevention and treatment of HIV. The misconceptions within the three coding themes were simultaneously explored.

### Results

#### Misconceptions about symptoms of HIV/AIDS

While correctly identifying that significant weight loss (or being slim) is a symptom of HIV/AIDS, adolescents expressed some mistaken ideas about changes in physical appearance that occur in HIV-infected persons. Some of these misconceptions include abdominal swelling, change in hair color, long finger nails, small head size and muscular paralyses (hemiplegia and paraplegia). There was also the general notion that HIV-infected persons can be easily recognized by how they look. These views about symptoms of HIV/AIDS were shared by both adolescent boys and girls.

*“The person has long fingernail… will be very slim, have big stomach and tiny legs. If you look at the person you will use your eyes to confirm that the person is infected by HIV/AIDs”* (Male Adolescent—ADIKM).

*“By mere looking at them you will know because they will be slim…. Also, HIV infected persons have stroke* (paralyses)*, they look very slim and the hairs of the person can change color.”* (Male Adolescent—ADEZM).

*“The head of an HIV*-*infected person will be very small and they look ugly and very tiny …”* (Female Adolescent—ADEZF).

Although HIV-related symptoms are commonly related to opportunistic infections, neurologic tropism is not a common symptom and it would take an advanced learner (such as a health worker) to identify (or mention) stroke as a symptom of HIV/AIDS.

The difference between weight loss and being slim could be hard to interpret from an adolescent’s point of view. Although HIV patients experience weight loss due to the disease, individuals could be naturally slim which is unrelated to HIV/AIDS.

#### Misconceptions about modes of transmission of HIV/AIDS

Some commonly reported misconceptions about modes of transmission of HIV/AIDS were seen to persist among adolescents, such as sharing the same bed; sharing personal belongings such as clothing, foot wear, combs, utensils and toothbrush; hugging and kissing; and sucking of breast. These ideas were also shared by adolescent boys or girls. Some supporting quotes include,

*“HIV can be contracted by using the same handkerchief with the person that has HIV. Also, through the comb that the HIV person used to comb his hair, and also wearing pants from one person to the other”* (Male Adolescent-ADEZM).

*“You contract HIV when a boy is sucking a breast of a girl that is HIV infected”* (Female Adolescent-ADABF).

*“You can contract it* (HIV) *when using spoon that somebody has used to eat and by sharing the same brush with another person who has the disease*” (Female Adolescent—ADEZFR).

#### Misconceptions about prevention and treatment of HIV

Compared to the other thematic areas, wrong impression about curability of HIV/AIDS was an exception rather than norm among adolescents that participated in the FGDs. Only a couple of male adolescents were of the opinion that HIV could be cured; one of them categorically stated that HIV could be cured using antibiotics and the other said he received his information about HIV cure from YouTube.

All other participants expressed that HIV has no cure but could be treated with antiretroviral drugs that reduce the viral load and contribute to improving the quality of life of HIV-infected persons. They also identified that HIV could be prevented through proper education, screening of blood before transfusion, and safe sex practices such as consistent use of condoms and sexual abstinence. Some supporting quotes are,

*“HIV can never be cured; but they (healthcare providers) can give you some drugs that can help to sustain your life”* (Female Adolescent-ADIKF).

*“HIV/AIDs patients who take drugs look healthy. They cannot be identified through their looks. Some of the boys that have HIV are even the healthiest persons in the community because of the use of antiretroviral drugs”* (Male Adolescent-ADAFM).

### Discussion

Efforts have been made to scale up HIV/AIDS treatment and prevention services for adolescents and young people [5]. The National HIV/AIDS Strategy for Adolescents and Young People (2016–2020) and National HIV and AIDS Strategic Framework (2019–2021) highlighted strategies for increasing community and individual knowledge about HIV using evidence- based information [6, 7]. A national sexuality education program was developed in Nigeria to reach a large population of young people in-school. However, due to strong criticisms from religious and other interest groups, the content of the program was revised and the current curriculum is reported to lack some important elements of comprehensive sexuality education such as prevention of HIV/AIDS [15].

Many adolescents in this study had correct knowledge of methods of prevention of HIV/AIDS. These adolescents were aware that there is no cure for the disease and that antiretroviral drugs work to reduce viral load and improve quality of life of HIV-infected people. However, there was a false belief that HIV/AIDS could be treated with antibiotics and misleading information about cure from the YouTube. The result of this study reveals that adolescents’ knowledge about HIV/AIDS is limited by persisting misconceptions about symptoms, modes of transmission and methods of prevention of HIV. Some studies have also reported that adolescents lack comprehensive and untainted knowledge of HIV/AIDS [4, 16]. These false beliefs influence adolescents’ views that HIV could be contracted from a healthy-looking person [16], and it increases vulnerability to HIV infection when unsuspecting adolescents engage in unprotected sexual intercourse with HIV-infected persons.

The fact that many adolescents could hardly identify correct modes of transmission of HIV, and that myths and misconceptions about HIV transmission persist among these adolescents is worrisome. Our findings corroborate studies that have reported false impressions that HIV/AIDS can be transmitted through casual contact with infected persons, mosquito bite, sharing of clothes, utensils, and direct contact such as hugging and kissing [16–18]. These findings validate assertions that behavioral change model which has been promoted in HIV education interventions does not sufficiently capture the influence of misconceptions in understanding HIV transmission [19, 20]. Hence, misconceptions about HIV transmission persist and are passed on from one generation to the next [19, 20].

Since misconceptions are a product of the interaction of personal, interpersonal, societal and cultural factors, dispelling these misconceptions would require refocusing efforts towards enhancing clinical knowledge of the modes of transmission of HIV among adolescents, and indeed all segments of the population [21, 22]. Many young people rely on the internet, which is loaded with lots of unverified advice, as a major source of information on health [23, 24]. Evidence shows that the highest demographic that accesses social media including YouTube channels comprise of adolescents and young people [13, 24]. As it may not be feasible to verify and censor all information posted on the internet, HIV program managers and implementers should use progressive and popular media platforms such as YouTube to provide correct information about HIV transmission and prevention to viewers.

## Conclusion

Several misconceptions about HIV/AIDS were reported by adolescents in this study, and these pose serious challenges to the control of HIV among this age group. The fact that some misconceptions about modes of transmission appear to be transferred from older to younger generations underscores the need to improve biological understandings of HIV transmission among adolescents and adults alike. This could be achieved by providing comprehensive HIV/AIDS education in schools through trained teachers, and in communities through local and conventional media. Revision of the already existing comprehensive HIV/AIDS education curriculum in school to include all components of HIV/AIDS education is also crucial. Well-designed interventions that will help in addressing misconceptions about HIV/AIDS among adolescents should be implemented in both urban and rural areas. Interventions using peer educators have proven to be effective, these programs should be supported to reduce HIV/AIDS misinformation among adolescents.

## Limitations

This was a qualitative research study that highlighted different misconceptions about HIV/AIDS among adolescents. As a qualitative study, it is limited in its ability to produce generalizable findings. Our findings could have been strengthened by a robust quantitative component which would estimate the magnitude of these misconceptions about HIV/AIDS among adolescents. However, the study findings present useful information that could be used in designing structured comprehensive questionnaires on adolescents’ sexual and reproductive health.

## Data Availability

Additional data from the research project could be made available by the corresponding author on reasonable request.

## Abbreviations

STIs: Sexually Transmitted Infections
HIV: Human Immunodeficiency virus
AIDS: Acquired Immunodeficiency Syndrome
FGDs: Focus Group Discussions
LGAs: Local Government Areas
IDRC: International Development Research Centre

## References

[1] UNICEF: UNAIDS, the Joint United Nations Programme on HIV/AIDS. Opportunity in Crisis: Preventing HIV from early adolescence to young adulthood. 2011. Retrieved from https://www.unicef.org/aids/files/Opportuinty in crisis-Report EN 052711(4).pdf. Accessed 2 Nov 2019.

[2] AVERT: HIV and AIDS in Nigeria: Global information and education on HIV and AIDS. 2019. https://www.avert.org/professionals/hiv-around-world/sub-saharan-africa/nigeria. Accessed 2 Nov 2019.

[3] Agaba PA, Makai R, Bankat CT, Chebu PR, Apena T, Iyaji-Paul O, Idoko JA (2016). Sexual behavior and risk factors for HIV infection among young people aged 15–24 years in North-Central Nigeria. J Med Tropics.

[4] National Population Commission (NPC) [Nigeria] and ICF International. Nigeria Demographic and Health Survey 2018. Abuja, Nigeria, and Rockville, Maryland, USA: NPC and ICF International. 2019. https://dhsprogram.com/pubs/pdf/PR118/PR118.pdf. Accessed 29 Nov 2019.

[5] UNAIDS: Press release. 2019. Retrieved 1 November 2019, from https://www.unaids.org/en/resources/presscentre/pressreleaseandstatementarchive/2019/march/20190314_nigeria. Accessed 29 Nov 2019.

[6] NACA. National HIV and AIDS Strategic Plan: 2017–2021. National agency for the control of AIDS. Federal Republic of Nigeria 2017. https://naca.gov.ng/national-guidelines-hiv-prevention-treatment-care-2/. Accessed 15 Nov 2019.

[7] NACA: National HIV Strategy for Adolescents and Young People: 2016–2020. 2016. https://naca.gov.ng/national-hiv-strategy-adolescents-young-people/. Accessed 17 Dec 2019.

[8] Van der Maas F, Otte MW (2009). Evaluation of HIV/AIDS secondary school peer education in rural Nigeria. Health Educ Res.

[9] UNAIDS: Global Report. 2013. http://files.unaids.org/en/media/unaids/contentassets/documents/epidemiology/2013/gr2013/UNAIDS_Global_Report_2013_en.pdf. Accessed 29 Nov 2019.

[10] Goris T, Dyrenfurth M. Students’ Misconceptions in Science, Technology and Engineering. 2010. https://pdfs.semanticscholar.org/73e9/7206ea56458c0ec7313871111ba37bcb63d8.pdf?_ga=2.80250711.172516139.1579007036-906000166.1541126681. Accessed 5 Jan 2020.

[11] Arnold R, Maticka-Tyndale E, Tenkorang E, Holland D, Gaspard A, Luginaah I, Team HP4RY. Evaluation of School- and Community-Based HIV Prevention Interventions with Junior Secondary School Students in Edo State, Nigeria. Afr J Reprod Health 2012; 16(2):103–125. https://www.ajol.info/index.php/ajrh/article/view/77842/68258. 22916547

[12] Amoyaw JA, Kuuire VZ, Boateng GO, Asare-Bediako Y, Ung M (2015). Conundrum of sexual decision making in marital relationships: safer-Sex knowledge, behavior, and attitudes of married women in Zambia. J Sex Res.

[13] Wong CA, Merchant RM, Moreno MA (2014). Using social media to engage adolescents and young adults with their health. Healthcare.

[14] USAID and Health Policy Plus (HP +). Nigeria Population and Development Ebonyi State. Abuja Nigeria 2017. http://www.healthpolicyplus.com/ns/pubs/7149-7286_EbonyiRAPIDFactSheet.pdf. Acessesed 9 Dec 2019.

[15] Wood SY; Rogow D: Can Sexuality Education Advance Gender Equality and Strengthen Education Overall? Learning from Nigeria’s Family Life and HIV Education Program. New York: International Women’s Health Coalition, October 2015. https://iwhc.org/wp-content/uploads/2015/12/Nigeria_FLHE_FINAL-nospreads.pdf. Accessed 23 Apr 2020.

[16] Singh A, Jain S (2009). Awareness of HIV/AIDS among school adolescents in Banaskantha district of Gujarat. Health Population Perspectives Issues.

[17] Deribew A, Abebe G, Apers L, Jira C, Tesfaye M, Shifa J, Abdisa A, Woldemicheal K, Deribie F, Bezabih M, Aseffa A, Colebunders R (2010). Prejudice and misconceptions about tuberculosis and HIV in rural and urban communities in Ethiopia: a challenge for the TB/HIV control program. BMC Public Health.

[18] Otokpa OA, Lawoyin OT, Asuzu CM (2013). HIV/AIDS-related knowledge and misconceptions among women attending government-owned antenatal clinics in Gwagwalada Area Council of Abuja, Nigeria. Afr J Reprod Health.

[19] Amuyunzu-Nyamongo M, Tendo-Wambua L, Babishangire B, Nyagero J, Yitbarek N, Matasha M, Omurwa T. Barriers to behaviour change as a response to STD including HIV/AIDS: the East African experience. Resistances to Behavioural Change to Reduce HIV/AIDS infection 1999; 1–11.

[20] Taylor JJ (2007). Assisting or compromising intervention? The concept of “culture” in biomedical and social research on HIV/AIDS. Soc Sci Med.

[21] Sano Y, Antabe R, Atuoye NK, Hussey KL, Bayne J, Galaa ZS, Mkandawire P, Luginaah I (2016). Persistent misconceptions about HIV transmission among males and females in Malawi. BMC Int Health and Human Rights.

[22] WHO. World Health Organization Fact Sheets: HIV/AIDS. 2019. https://www.who.int/news-room/fact-sheets/detail/hiv-aids. Accessed 15 Nov 2019.

[23] Diviani N, van den Putte B, Giani S, van Weert JC (2015). Low health literacy and evaluation of online health information: a systematic review of the literature. J Med Internet Res.

[24] Villanti AC, Johnson AL, Ilakkuvan V, Jacobs MA, Graham AL, Rath JM (2017). Social media use and access to digital technology in US young adults in 2016. J Med Internet Res.

